# *In Vitro* Enzymatic Depolymerization of Lignin with Release of Syringyl, Guaiacyl, and Tricin Units

**DOI:** 10.1128/AEM.02076-17

**Published:** 2018-01-17

**Authors:** Daniel L. Gall, Wayne S. Kontur, Wu Lan, Hoon Kim, Yanding Li, John Ralph, Timothy J. Donohue, Daniel R. Noguera

**Affiliations:** a Great Lakes Bioenergy Research Center, Wisconsin Energy Institute, University of Wisconsin, Madison, Wisconsin, USA; b Department of Biochemistry, University of Wisconsin, Madison, Wisconsin, USA; c Department of Bacteriology, University of Wisconsin, Madison, Wisconsin, USA; d Department of Civil & Environmental Engineering, University of Wisconsin, Madison, Wisconsin, USA; North Carolina State University

**Keywords:** depolymerization, guaiacyl, Lig pathway, lignin, sphingmonads, syringyl, tricin

## Abstract

New environmentally sound technologies are needed to derive valuable compounds from renewable resources. Lignin, an abundant polymer in terrestrial plants comprised predominantly of guaiacyl and syringyl monoaromatic phenylpropanoid units, is a potential natural source of aromatic compounds. In addition, the plant secondary metabolite tricin is a recently discovered and moderately abundant flavonoid in grasses. The most prevalent interunit linkage between guaiacyl, syringyl, and tricin units is the β-ether linkage. Previous studies have shown that bacterial β-etherase pathway enzymes catalyze glutathione-dependent cleavage of β-ether bonds in dimeric β-ether lignin model compounds. To date, however, it remains unclear whether the known β-etherase enzymes are active on lignin polymers. Here we report on enzymes that catalyze β-ether cleavage from bona fide lignin, under conditions that recycle the cosubstrates NAD^+^ and glutathione. Guaiacyl, syringyl, and tricin derivatives were identified as reaction products when different model compounds or lignin fractions were used as substrates. These results demonstrate an *in vitro* enzymatic system that can recycle cosubstrates while releasing aromatic monomers from model compounds as well as natural and engineered lignin oligomers. These findings can improve the ability to produce valuable aromatic compounds from a renewable resource like lignin.

**IMPORTANCE** Many bacteria are predicted to contain enzymes that could convert renewable carbon sources into substitutes for compounds that are derived from petroleum. The β-etherase pathway present in sphingomonad bacteria could cleave the abundant β–O–4-aryl ether bonds in plant lignin, releasing a biobased source of aromatic compounds for the chemical industry. However, the activity of these enzymes on the complex aromatic oligomers found in plant lignin is unknown. Here we demonstrate biodegradation of lignin polymers using a minimal set of β-etherase pathway enzymes, the ability to recycle needed cofactors (glutathione and NAD^+^) *in vitro*, and the release of guaiacyl, syringyl, and tricin as depolymerized products from lignin. These observations provide critical evidence for the use and future optimization of these bacterial β-etherase pathway enzymes for industrial-level biotechnological applications designed to derive high-value monomeric aromatic compounds from lignin.

## INTRODUCTION

There is economic and environmental interest in using renewable resources as raw materials for production of chemicals that are currently derived from fossil fuels. Lignin, a renewable resource that accounts for ∼15 to 30% (dry weight) of vascular plant cell walls (1, 2), is comprised of aromatic compounds that may be valuable commodities for the biofuel, chemical, cosmetic, food, and pharmaceutical industries (3). Consequently, intensive efforts are currently aimed at developing chemical, enzymatic, and hybrid methods for deriving simpler and lower-molecular-weight (lower-MW) products from lignin (4).

**FIG 1:**
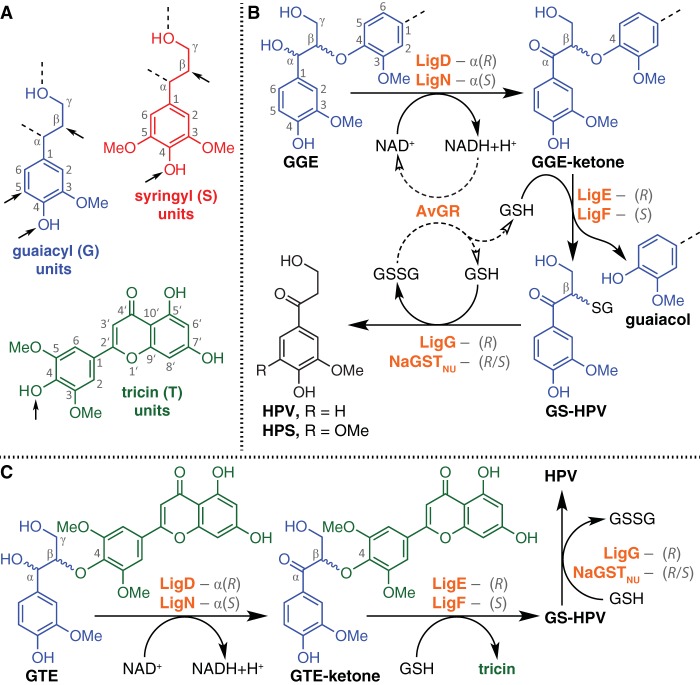
Aromatic monomers and the β-etherase pathway. Panel A shows the structures of predominant monomeric phenylpropanoids found in lignin, guaiacyl (G, in blue), syringyl (S, in red), and tricin (T, in green) units. Arrows indicate where interunit linkages are formed during radical coupling reactions. Dashed lines indicate positions that may form additional covalent bonds during postcoupling reaction mechanisms. Panel B shows β-etherase pathway-mediated degradation of the diaromatic β-ether-linked model compound GGE via NAD^+^-dependent dehydrogenases LigD and LigN to form GGE-ketone and NADH. GGE-ketone undergoes GSH-dependent β-ether cleavage by β-etherase enzymes LigE and LigF to yield guaiacol and GS-HPV as monoaromatic derivative products. GS-HPV undergoes GSH-dependent thioether cleavage by NaGST_NU_ or LigG, producing GSSG and monoaromatic product HPV. As indicated by the dashed arrows, AvGR recycles cosubstrates GSH and NAD^+^ via NADH-dependent reduction of GSSG. For reactions involving an *R*- or *S*-configured epimer as the substrate, the isomer toward which each enzyme exhibits activity is shown in gray text. Panel C shows how β-etherase pathway enzymes degrade GTE through intermediate GTE-ketone to yield tricin and HPV.

The lignin backbone is predominantly composed of guaiacyl (G) and syringyl (S) phenylpropanoid units (Fig. 1A) that derive from the monomers coniferyl alcohol and sinapyl alcohol, which become covalently linked during lignification via radical coupling reactions, primarily by endwise addition of a monomer (radical) to the phenolic end of the growing polymer (radical). G and S units are interlinked by a variety of chemical bonds by which the units are characterized: resinols (β–β), 4–O–5-diaryl ethers, phenylcoumarans (β–5), and β–O–4-aryl ethers (termed β-ethers here) (5–7). In grasses, the flavone tricin (T unit [Fig. 1A]) begins a chain and is covalently linked to the next unit via a 4–O–β-ether bond (8–10). Given that approximately 50 to 70% of all interunit linkages in lignin are β-ethers (5–7), cleavage of these bonds is crucial for processes aiming to derive valuable low-molecular-weight compounds from lignin in high yields. The formation of β-ether linkages during lignification generates a racemic lignin product containing both β(*R*)- and β(*S*)-carbons that, after rearomatization of the quinone methide intermediate by proton-assisted water addition, are adjacent to either α(*R*)- or α(*S*)-configured benzylic alcohols (11–13). Each unit therefore has 4 optical isomers and two “real” isomers—the so-designated *threo* and *erythro* (or *syn* and *anti*) isomers. Lignin depolymerization via β-ether bond cleavage has been demonstrated with chemical catalysis (14, 15). In addition, cytoplasmic enzymes in a sphingomonad β-etherase pathway have been identified that oxidize and cleave model β-ether-linked aromatic dimers (16).

The β-etherase pathway is present in Sphingobium sp. strain SYK-6 and other sphingomonads (e.g., Novosphingobium spp.) (16, 17). The diaromatic β-ether-linked guaiacylglycerol-β-guaiacyl ether (GGE [Fig. 1B]) lignin model compound has been used as a substrate to identify the following three enzymatic steps in cleavage of β-ether linkages *in vitro* (17–21): (i) a set of dehydrogenases catalyze NAD (NAD^+^)-dependent α-oxidation of GGE to GGE-ketone, also referred to as α-(2-methoxyphenoxy)-β-hydroxypropiovanillone (MPHPV), and NADH (19, 22); (ii) β-etherases, members of the glutathione *S*-transferase (GST) superfamily, carry out glutathione (GSH)-dependent cleavage of GGE-ketone, releasing guaiacol and β-S-glutathionyl-γ-hydroxypropiovanillone (GS-HPV) (18, 21, 23); and (iii) one or more glutathione lyases catalyze GSH-dependent cleavage of GS-HPV, yielding glutathione disulfide (GSSG) and γ-hydroxypropiovanillone (HPV) (18, 21, 24; W. S. Kontur, C. Bingman, C. Olmstead, D. Wassarman, A. Ulbrich, D. L. Gall, R. W. Smith, L. M. Yusko, B. G. Fox, D. R. Noguera, J. J. Coon, and T. J. Donohue, submitted for publication).

The use of multiple enzymes for some of the pathway's steps is attributable to the existence of both *R*- and *S*-configured chiral centers in lignin (11–13). The known NAD^+^-dependent dehydrogenases (LigD, LigL, LigN, and LigO) exhibit strict stereospecificity at the α position, with indifference to the configuration at the β position (19). With model diaromatic substrates, LigD and LigO are active on the *R*-configured α-epimers, whereas LigL and LigN are active on the *S*-configured α-epimers. Because the combined activity of these dehydrogenases eliminates the chiral center at α, GGE-ketone exists as two β-enantiomers that are cleaved by stereospecific β-etherases LigE, LigP, and LigF, each of which catalyzes the release of guaiacol with chiral inversion at the β position, and one of two β-epimers of GS-HPV [LigE and LigP convert β(*R*)-GGE-ketone to β(*S*)-GS-HPV, and LigF converts β(*S*)-GGE-ketone to β(*R*)-GS-HPV] (21). The final step is the GSH-dependent cleavage of the GS-HPV epimers, yielding GSSG and HPV as coproducts. LigG has been shown to cleave both β(*R*)-GS-HPV and β(*S*)-GS-HPV (24), although it appears to have a strong preference for the former (18, 21). Recently, a GSH transferase from Novosphingobium aromaticivorans DSM12444 (NaGST_NU_; Saro_2595 in GenBank assembly GCA_000013325.1) (Kontur et al., submitted) has been shown to have high activity with β(*R*)-GS-HPV and β(*S*)-GS-HPV both *in vivo* and *in vitro*, producing HPV and GSSG as products (Fig. 1B).

Despite what is known about the activity of individual β-etherase pathway enzymes with model diaromatic compounds, there is little information on their function with lignin oligomers. *In vivo* activity may be limited to aromatic dimers or small lignin oligomers due to restrictions in transporting large polymers into the bacterial cytoplasm, where the β-etherase pathway enzymes are found. Recently, modest recovery of low-molecular-mass aromatic compounds from lignin from a multistep enzymatic process that used a laccase mediator system, two β-etherases, and a glutathione lyase was reported (25). However, the size of lignin fragments that were subject to enzymatic cleavage was not determined, and it therefore remains unknown whether the β-etherases were active on only small or also large lignin oligomers.

To better understand the function of β-etherase pathway enzymes, we sought to use a minimal set of enzymes to develop a coupled *in vitro* assay capable of releasing G, S, and T aromatic monomers and recycling the cosubstrates NAD^+^ and GSH. Here we demonstrate complete conversion of GGE to guaiacol and HPV in a reaction including LigD, LigN, LigE, LigF, NaGST_NU_, and the Allochromatium vinosum DSM180 GSH reductase (AvGR), which catalyzes NADH-dependent reduction of GSSG and recycles the cofactors needed in the reaction (Fig. 1B) (26). We also show that this combination of enzymes releases tricin from the model compound guaiacylglycerol-β-tricin ether (GTE [Fig. 1C]). In addition, we show that the same combination of enzymes releases G, S, and T units from bona fide lignin oligomers. We discuss new insights gained from this study and its implications for the future production of these and possibly other valuable products from lignin.

## RESULTS

### Design of a coupled *in vitro* assay for cleavage of β-ether-linked diaromatic compounds.

As an initial substrate for this assay we used *erythro*-GGE, which is a mixture of enantiomers (α*R*,β*S*)-GGE and (α*S*,β*R*)-GGE that has been used extensively as a substrate with β-etherase pathway enzymes (17–21). We used recombinant preparations of LigD and LigN, as these dehydrogenases are reported to be sufficient for the NAD^+^-dependent oxidation of *R*- and *S*-configured α anomers of *erythro*-GGE *in vitro* (19). The assay also contained recombinant preparations of LigE and LigF that have been shown to separately catalyze the GSH-dependent conversion of a racemic mixture of GGE to guaiacol and the *S*- and *R*-epimers of GS-HPV (21). NaGST_NU_ was present to catalyze the GSH-dependent cleavage of the GS-HPV epimers to HPV and GSSG. The properties of individual enzymes (Fig. 1B) predict that this coupled system will require equimolar concentrations of GGE and NAD^+^ and twice as much GSH for complete conversion of GGE to HPV and guaiacol.

**FIG 2:**
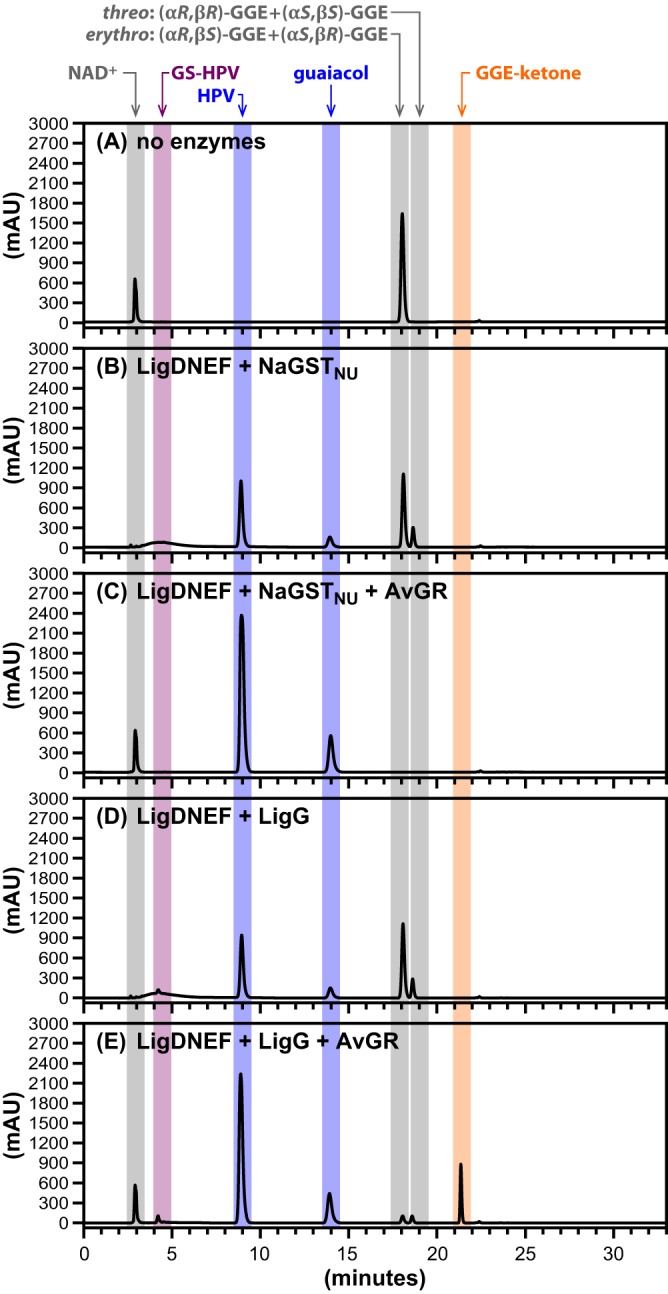
HPLC traces of substrates and products of β-etherase pathway assays using NAD^+^ (2.0 mM), GSH (4.0 mM), and *erythro*-GGE (6.0 mM). Elution times of compounds (absorbance at 280 nm) are highlighted by colored bands for NAD^+^ and NADH (∼3.0 min), GS-HPV (∼4.5 min), HPV (∼9.0 min), guaiacol (∼14.0 min), *erythro*-GGE (∼18.0 min), *threo*-GGE (∼19.0 min), and GGE-ketone (∼21.5 min). Structures of GS-HPV, HPV, guaiacol, GGE, and GGE-ketone are shown in Fig. 1B. Panel A shows a control sample to which no enzymes were added. The remaining panels show products in assays containing LigDNEF and NaGST_NU_ (B), LigDNEF, NaGST_NU_, and AvGR (C), LigDNEF and LigG (D), and LigDNEF, LigG, and AvGR (E) after 4 h of incubation of these combinations of enzymatic catalysts (50 μg/ml of each).

In an attempt to reduce the amount of added NAD^+^ and GSH that would be needed for full conversion of diaromatic substrate to products, some reaction mixtures included recombinant AvGR, which catalyzes the NADH-dependent reduction of GSSG (26), thereby recycling the cosubstrates NAD^+^ and GSH for continued conversion of the β-ether substrates. This cosubstrate recycling system was tested with 6 mM *erythro*-GGE and limiting concentrations of NAD^+^ (2 mM) and GSH (4 mM) (Fig. 2A). For a summary of all reactions carried out in this study, see Table S1 in the supplemental material. Using a mixture of LigD, LigN, LigE, LigF, and NaGST_NU_, without AvGR (reaction 1 in Table S1), we observed that *erythro*-GGE was partially converted to HPV and guaiacol (Fig. 2B). Quantification of this assay revealed that the *erythro*-GGE concentration decreased from 6.0 mM to 3.8 mM at the end of the assay, whereas the HPV and guaiacol concentrations were each 2.0 mM, the NAD^+^ levels were nondetectable, and the concentration of *threo*-GGE [a mixture of enantiomers (α*R*,β*R*)-GGE and (α*S*,β*S*)-GGE] increased to 0.1 mM, presumably due to the reported reversibility of the LigD/LigN reactions (27). Thus, the final GGE concentration (3.9 mM, the sum of the *erythro*-GGE and *threo*-GGE concentrations) was consistent with consumption of 2.0 mM NAD^+^. In addition, the production of 2.0 mM (each) HPV and guaiacol was consistent with the consumption of 4.0 mM GSH, where 2.0 mM GSH was consumed in the LigE/LigF reactions and an additional 2.0 mM GSH was consumed in the NaGST_NU_ reaction.

**FIG 3:**
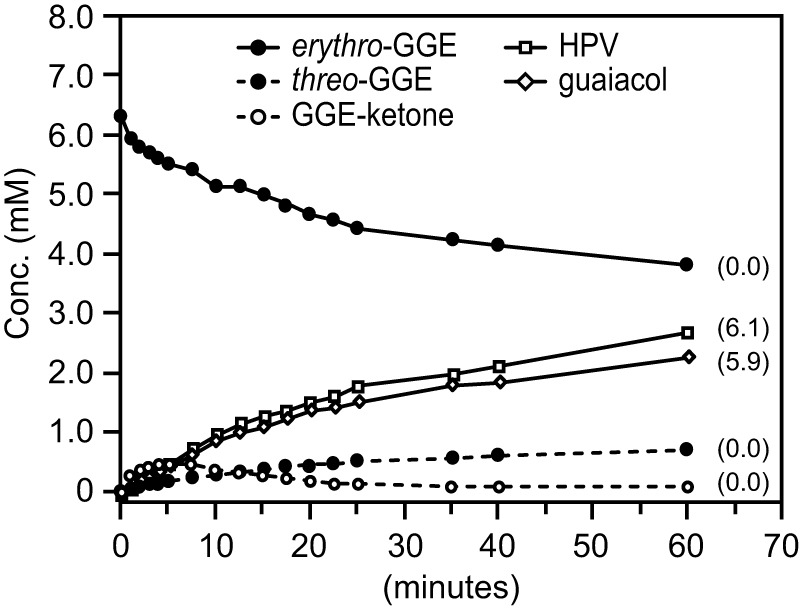
Time-dependent changes in concentrations of *erythro*-GGE, *threo*-GGE, GGE-ketone, HPV, and guaiacol in an assay mixture that, at 0 min, was supplemented with NAD^+^ (2.0 mM), GSH (4.0 mM), and *erythro*-GGE (6.0 mM), as well as (50 μg/ml each) LigD, LigN, LigE, LigF, NaGST_NU_, and AvGR. Numbers in parentheses represent the measured concentration (millimolar) of each compound after 4 h of incubation. Structures of HPV, guaiacol, GGE, and GGE-ketone are shown in Fig. 1B. *Erythro*-GGE is a mixture of enantiomers (α*R*,β*S*)-GGE and (α*S*,β*R*)-GGE. *Threo*-GGE is a mixture of enantiomers (α*S*,β*S*)-GGE and (α*R*,β*R*)-GGE.

To test the impact of AvGR on this assay, we added it to a parallel *in vitro* reaction (reaction 2 in Table S1). We found that in the presence of AvGR (Fig. 2C), GGE was completely consumed and equimolar amounts of HPV and guaiacol (6.0 mM each) were produced, without a detectable change in the NAD^+^ concentration or accumulation of any β-etherase pathway intermediates by the time of the assay's conclusion. To determine if any β-etherase pathway intermediates accumulated over the course of the assay, we tested for time-dependent changes in the concentrations of the substrate, known pathway intermediates, and products in a parallel reaction (Fig. 3). We found that as *erythro*-GGE degradation occurs there is a time-dependent accumulation and depletion of GGE-ketone and *threo*-GGE and, eventually, complete equimolar conversion of the substrate to HPV and guaiacol (Fig. 2A to C). From these results, we conclude that the combination of LigD, LigN, LigE, LigF, NaGST_NU_, and AvGR is sufficient to process all of the chiral centers in a β-ether substrate such as *erythro*-GGE. In addition, we conclude that the presence of AvGR is sufficient to recycle the cosubstrates NAD^+^ and GSH that are needed for cleavage of β-ether bonds in a model diaromatic compound such as *erythro*-GGE.

From information available in the literature, it has remained unclear whether the GSH lyase from Sphingobium strain SYK-6, LigG, has a preference for β(*R*)-GS-HPV (18, 21) or is capable of cleaving the thioether linkages in both β(*R*)-GS-HPV and β(*S*)-GS-HPV (24). As the presence of NaGST_NU_ resulted in cleavage of both β(*R*)-GS-HPV and β(*S*)-GS-HPV in this coupled reaction system (Fig. 2A to C), we sought to use this *in vitro* assay to test the activity of LigG under identical conditions (reaction 3 in Table S1). When we performed an assay using 6.0 mM *erythro*-GGE, 2.0 mM NAD^+^, and 4.0 mM GSH, as well as the mixture of LigD, LigN, LigE, LigF, and LigG (without AvGR), we observed partial conversion of GGE to HPV and guaiacol (Fig. 2D). At the end of this assay, the total GGE concentration (4.0 mM, the sum of the *erythro*-GGE and *threo*-GGE concentrations) was expected based on the consumption of 2.0 mM NAD^+^. Further, the production of 2.0 mM (each) HPV and guaiacol was consistent with the consumption of 4.0 mM GSH (2.0 mM GSH consumed by each of the LigE/LigF and LigG reaction steps). When we added AvGR to a parallel reaction (reaction 4 in Table S1) mixture that contained GGE (6.0 mM), NAD^+^ (2 mM), GSH (4 mM), and a combination of LigD, LigN, LigE, LigF, and LigG, we did not observe complete conversion of GGE to HPV and guaiacol (Fig. 2E). Instead, we detected the diaromatic substrate (*erythro*-GGE), *threo*-GGE, GS-HPV, and GGE-ketone (0.7 mM). In contrast to what was found when NaGST_NU_ was present under identical reaction conditions, the presence of LigG led to incomplete utilization of the diaromatic substrate and the accumulation of β-etherase pathway intermediates. From these results, we conclude that LigG is not able to completely cleave both β-epimers of GS-HPV *in vitro*. Consequently, all subsequent assays were performed using NaGST_NU_ as a source of GSH lyase activity.

### Production of tricin from GTE *in vitro*.

**FIG 4:**
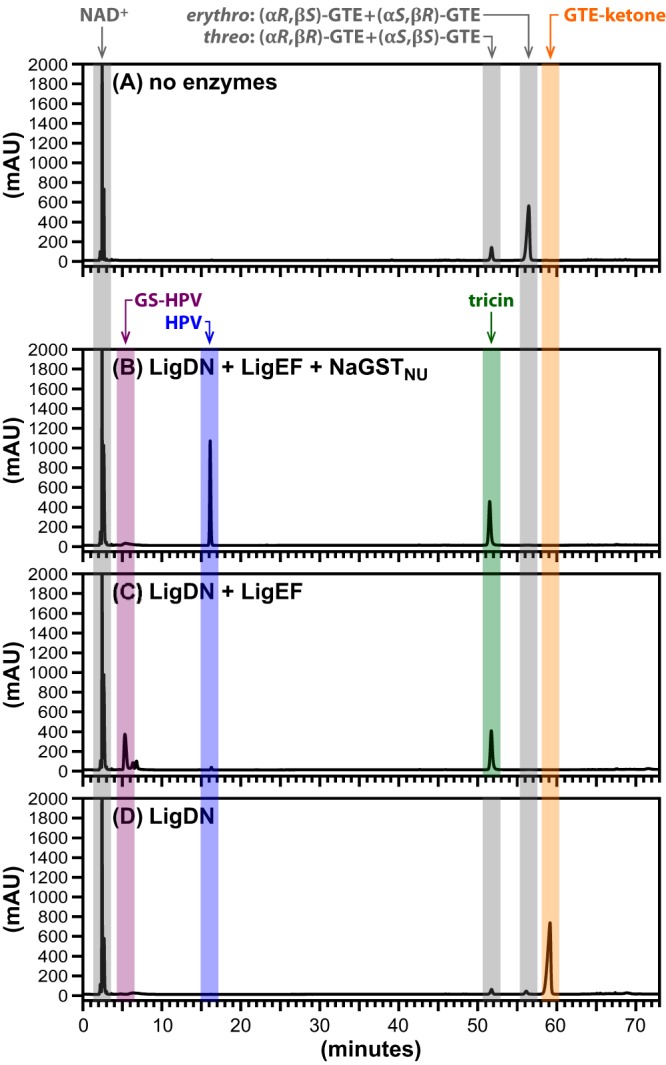
HPLC traces of β-etherase pathway products in reactions including the indicated enzymes and NAD^+^ (5.0 mM), GSH (5.0 mM), and GTE (1.0 mM; a 6:1 mixture of *erythro*-GGE to *threo*-GTE). Elution times of compounds (absorbance at 280 nm) are highlighted by colored bands for NAD^+^ and NADH (∼3.0 min), GS-HPV (∼5.5 min), HPV (∼16.5 min), tricin (∼51.5 min), *threo*-GTE (∼52.0 min), *erythro*-GTE (∼56.0 min), and GTE-ketone (∼58.5 min). Panel A shows the control sample, to which no enzymes were added. The remaining panels show products in assays containing LigD, LigN, LigE, LigF, and NaGST_NU_ (B), LigD, LigN, LigE and LigF (C), and LigD and LigN (D) after 4 h of incubation of these combinations of enzymatic catalysts (50 μg/ml each). Structures of GS-HPV, HPV, tricin, GTE, and GTE-ketone are shown in Fig. 1.

In grasses, the flavone tricin (T [Fig. 1A]) is covalently linked to one end of lignin, via a β-ether bond (8, 9, 28). Although β-etherase pathway enzymes have been shown to cleave β-ether-linked diaromatic model compounds containing G and S monomers, to date there are no published data on their ability to remove the diaromatic flavonoid T units from any substrate. Thus, we sought to test the ability of the coupled assay to cleave GTE (Fig. 1C), a model compound containing a β-ether-linked tricin moiety. High-performance liquid chromatography (HPLC) analysis of the synthetic GTE (Fig. 4A) indicated that it contained a 6:1 ratio of *erythro*-GTE [(α*R*,β*S*)-GTE and (α*S*,β*R*)-GTE] to *threo*-GTE [(α*R*,β*R*)-GTE and (α*S*,β*S*)-GTE], which was consistent with the nuclear magnetic resonance (NMR) analysis of this material (8).

When we incubated 1.0 mM GTE, 5.0 mM NAD^+^, and 5.0 mM GSH with the combination of LigD, LigN, LigE, LigF and NaGST_NU_ (reaction 5 in Table S1), we observed the complete conversion of GTE to tricin and HPV (Fig. 4B). This result predicts that LigD and LigN oxidize GTE to form GTE-ketone, LigE and LigF catalyze β-ether cleavage in GTE-ketone to form GS-HPV and tricin, and NaGST_NU_ releases HPV from GS-HPV (Fig. 1C), suggesting that the larger β-ether-linked flavone model was able to access the active sites in LigD, LigN, LigE, and LigF. To test this hypothesis, we assayed for the presence of the expected β-etherase pathway intermediates, GS-HPV and GTE-ketone, from GTE. By performing a parallel reaction including the same substrates and only LigD, LigN, LigE, and LigF (reaction 6 in Table S1), we observed that GTE was degraded and tricin was produced (Fig. 4C). However, in this assay, there was no detectable production of HPV and we observed accumulation of GS-HPV. These findings indicate that the absence of NaGST_NU_ prevented the conversion of GS-HPV to HPV (Fig. 1C). Finally, in an assay including only the enzymes LigD and LigN (reaction 7 in Table S1), we found that GTE was almost completely converted to GTE-ketone (Fig. 4D), as expected from the NAD^+^-dependent α-oxidation of GTE. Together, the data show that T units can be derived from β-ether-linked model compounds *in vitro* using enzymes, cosubstrates, and intermediates that are known to be part of the β-etherase pathway (Fig. 1).

### Release of G, S, and T units from lignin oligomers.

**FIG 5:**
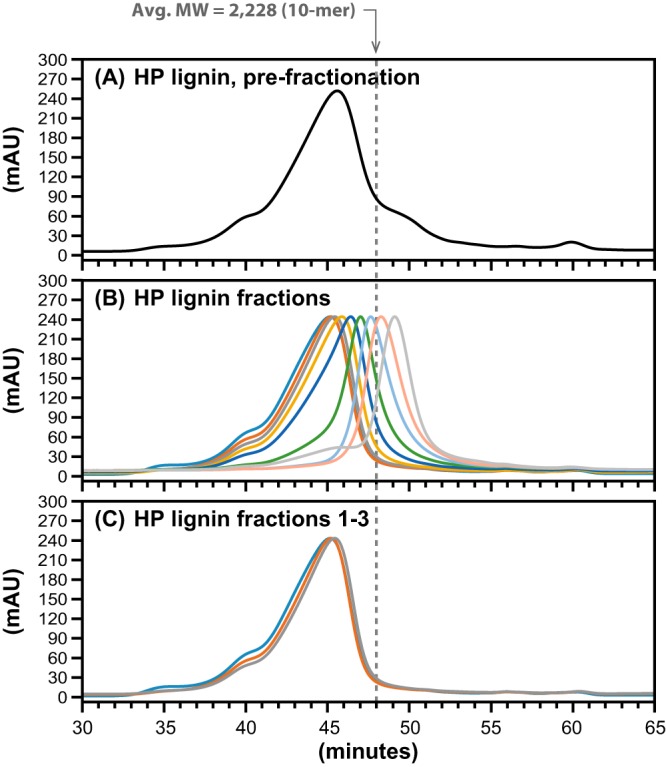
Analytical GPC traces (λ = 200 nm) showing the size distribution of unfractionated HP lignin (MW = 8,665) (A), fractions of HP lignin collected from preparative GPC (B), and the fractions that were pooled and used as the substrate in enzyme assays: fraction 1 (MW = 11,550), fraction 2 (MW = 10,780), and fraction 3 (MW = 9,340) (C). For reference, the approximate MW of a 10-mer is indicated with a dashed line.

**FIG 6:**
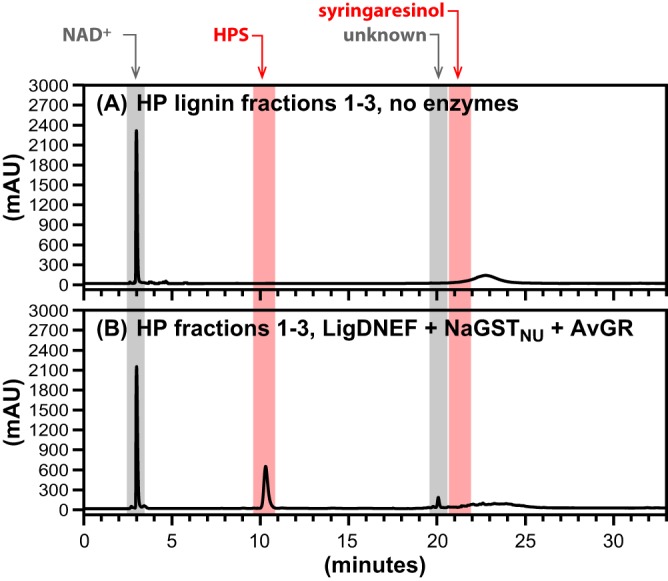
HPLC traces of coupled β-etherase pathway reactions supplemented with NAD^+^ (2.0 mM), GSH (4.0 mM), and HP lignin fractions (2.2 mg ml^−1^). Elution times of compounds (absorbance at 280 nm) are highlighted by colored bands for NAD^+^ and NADH (∼3.0 min), HPS (∼10.0 min), an unknown (∼20.0 min), and syringaresinol (∼21.5 min). Panel A shows the pooled GPC fractions 1 (MW = 11,550), 2 (MW = 10,780), and 3 (MW = 9,340) without enzyme addition. Panel B shows products after 4 h of incubation with LigD, LigN, LigE, LigF, NaGST_NU_, and AvGR (50 μg/ml each) and pooled HP lignin fractions 1 to 3 as the substrate.

With the coupled enzymatic system in place, we tested it for activity with lignin oligomers. First, we tested if a mixture of LigD, LigN, LigE, LigF, NaGST_NU_, and AvGR (reaction 8 in Table S1) produced S units from a high-syringyl hybrid poplar (HP) lignin polymer (29, 30). To ensure that the test was performed with lignin oligomers rather than low-MW material present in the lignin, we fractionated the HP lignin by gel permeation chromatography (GPC) and pooled the high-MW fractions (Fig. 5; Table 1) for use as a substrate (Fig. 6). From 2.2 mg ml^−1^ of lignin oligomers having MWs between 9,000 and 12,000 (with 2 mM NAD^+^ and 4 mM GSH), we detected the production of 1.0 mM γ-hydroxypropiosyringone (HPS), the HPV analog expected to be produced by cleavage of β-ether bonds from a syringyl unit at one end of the lignin chain. We also detected the formation of an unknown product in this reaction (Fig. 6B), which could be either a chemically modified S unit released from the HP lignin or a GS-linked intermediate product. Furthermore, syringaresinol, a dimeric unit in the HP lignin polymer (29), was not detected as a product of the enzymatic reaction.

**TABLE 1:** Estimated sizes of the HP lignin fractions after preparative GPC

Sample or fraction^*a*^	Avg MW^*b*^	Avg length (U)^*c*^
Original sample, prefractionation	8,665	38.3
Fraction 1*	11,550	51.0
Fraction 2*	10,780	47.6
Fraction 3*	9,340	41.3
Fraction 4	7,240	32.0
Fraction 5	5,200	23.0
Fraction 6	3,720	16.5
Fraction 7	2,660	11.8
Fraction 8	1,910	8.5
Fraction 9	1,280	5.8

a Asterisks indicate fractions that were pooled and used as the substrate in enzyme assays.

b Size was determined by analytical GPC.

c Polymer length is reported in number of units, based on the MWs of syringaresinol (418.44) and β-ether-linked syringyl units (228.24).

**FIG 7:**
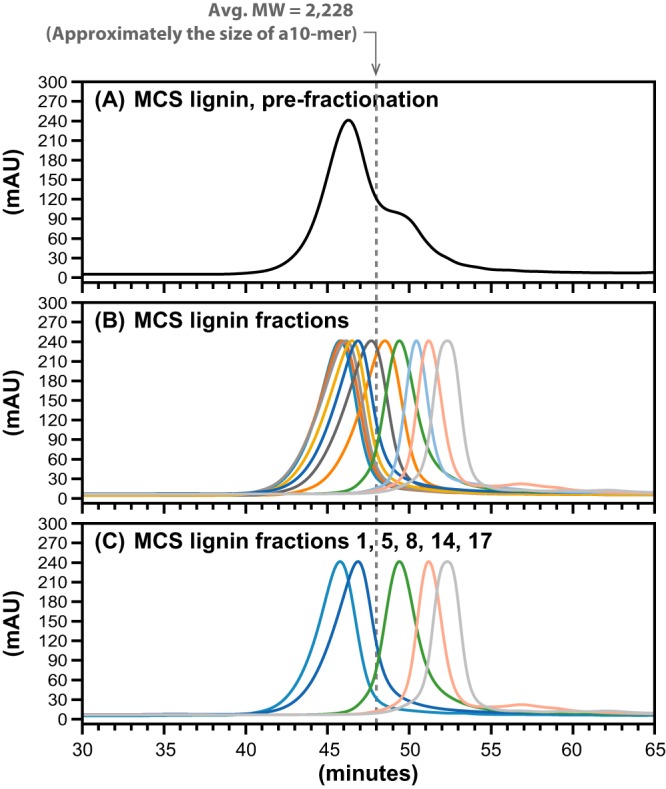
Analytical GPC traces (λ = 200 nm) showing the distribution of unfractionated MCS lignin (MW = 5,980) (A), fractions of MCS lignin collected from preparative GPC (B), and the fractions used as substrates in enzyme assays: fraction 1 (MW = 10,710), fraction 5 (MW = 5,370), fraction 8 (MW = 1,390), fraction 14 (MW = 660), and fraction 17 (MW = 460) (C). For reference, the approximate MW of a 10-mer is indicated with a dashed line.

**FIG 8:**
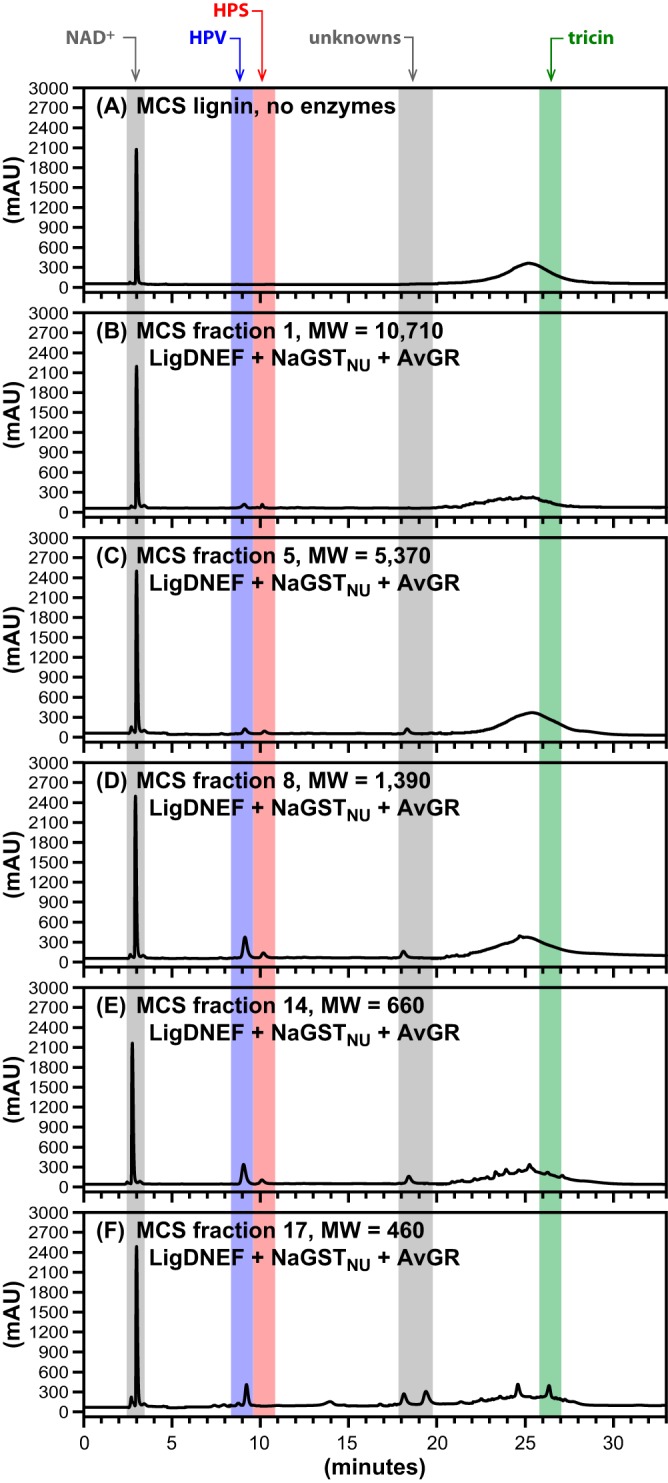
HPLC traces of β-etherase pathway enzyme activities in reactions including NAD^+^ (2.0 mM), GSH (4.0 mM), and MCS lignin or the indicated MCS lignin fractions (2.2 mg ml^−1^). Elution times (absorbance at 280 nm) are highlighted by colored bands for NAD^+^ (∼3.0 min), HPV (∼9.0 min), HPS (∼10.0 min), unknowns (∼18.0 to 19.0 min), and tricin (∼26.5 min) and an unknown broad peak (∼22.0 to 29.0 min); although this broad peak overlaps the tricin region, the authentic tricin peak is sufficiently sharp (especially in panel F) that its detection (and authentication) is not an issue. Panel A shows the control sample (unfractionated by GPC), to which no enzymes were added. The remaining panels show products after 4 h of incubation with 50 μg/ml each of LigD, LigN, LigE, LigF, NaGST_NU_ and AvGR and one of the following MCS lignin fractions: fraction 1 (MW = 10,710) (B), fraction 5 (MW = 5,370) (C), fraction 8 (MW = 1,390) (D), fraction 14 (MW = 660) (E), and fraction 17 (MW = 460) (F). Structures of HPV, HPS, and tricin are shown in Fig. 1.

Given the ability of the enzymatic assay to release HPS from HP lignin, we also tested for the release of aromatic monomers from a more complex lignin, such as the one derived from maize corn stover (MCS) (8, 9). To generate substrates for these assays, we used preparative GPC to size fractionate MCS lignin (Fig. 7; Table 2) and tested materials with different apparent MWs as the source of lignin oligomer substrates for enzyme assays (Fig. 8). To test for activity with these samples, we incubated LigD, LigN, LigE, LigF, NaGST_NU_, and AvGR (reactions 9 to 13 in Table S1) with MCS lignin oligomers (2.2 mg ml^−1^), 2.0 mM NAD^+^, and 4.0 mM GSH (Fig. 8A). In these experiments, we detected release of HPV and HPS in assays using lignin oligomers with average MW ranging from 460 to 10,710 (Fig. 7). The highest concentrations of HPV (0.4 mM) and HPS (0.1 mM) were observed with lignin oligomers having an average MW of 1,390 (Fig. 8D) as the substrate. In general, larger lignin oligomers resulted in lower accumulation of HPV and HPS. In addition, similar to the observations with HP lignin, unknown products were detected in most of the enzymatic reactions with the different lignin fractions (Fig. 8). Tricin was observed as a reaction product only when using the lowest-MW fraction tested (MW = 460 [Fig. 8F]). In sum, we conclude from these experiments that a combination of LigD, LigN, LigE, LigF, NaGST_NU_, and AvGR can release some, but not all, G, S, and T units from MCS lignin oligomers.

**TABLE 2:** Estimated size of the MCS lignin fractions after preparative GPC

Sample or fraction^*a*^	Avg MW^*b*^	Avg length (U)^*c*^
Original sample, prefractionation	5,980	28.5
Fraction 1*	10,710	51.0
Fraction 2	9,860	46.9
Fraction 3	8,320	39.6
Fraction 4	6,690	31.9
Fraction 5*	5,370	25.6
Fraction 6	3,930	18.7
Fraction 7	2,110	10.1
Fraction 8*	1,390	6.6
Fraction 11	880	4.2
Fraction 14*	660	3.1
Fraction 17*	460	2.2

a Asterisks indicate fractions that were used as the substrate in enzyme assays.

b Size was determined by analytical GPC.

c Polymer length is reported in number of units, based on the crude assumption that the average unit has an MW of 210.

## DISCUSSION

In order to use a polymer like lignin as a source of valuable aromatics and other chemicals, it is necessary to develop new or improve on existing depolymerization strategies. Recently developed oxidative and hydrogenolytic depolymerization approaches are showing some promise for producing low-molecular-weight aromatics in near-theoretical yields (15, 30), but there has also been considerable interest in exploring the biological production of aromatics from this renewable plant polymer (25). The use of the bacterial β-etherase pathway for biological depolymerization involves milder conditions that could result in the release of monomers, such as HPS and HPV, that are useful in their own right or extractable low-MW mixtures that could be further valorized (25, 31). There is now a large amount of information on the types of model diaromatic substrates recognized by individual β-etherase enzymes *in vitro*, the products of their activity, and their structural or functional relationships to other known enzymes (27, 32). Despite this, information on their activity with lignin oligomers is lacking. In addition, as these are cytoplasmic enzymes, it is plausible that they evolved to break down β-ether links only in the smaller lignin oligomers that could be transported inside the cells. A recent study described an *in vitro* enzymatic treatment of lignin yielding 12% (by weight) of low-molecular-mass aromatic products, although it remains unknown whether these aromatics were released from only small or also large oligomers (25).

In this work, we sought to develop a coupled *in vitro* system containing a set of β-etherase pathway enzymes that was capable of releasing monoaromatic compounds when incubated with different substrates. We reasoned that such a system would provide additional information on the β-etherase enzymes and aid in studies aimed at determining the requirements for release of valuable aromatics from bona fide lignin oligomers. We identified a minimum set of enzymes (LigD, LigN, LigE, LigF, NaGST_NU_, and AvGR) that, in a single procedure, cleaves β-ether linkages and completely converts model diaromatic compounds to aromatic monomers. We further showed that this coupled *in vitro* assay system is capable of stoichiometric production of monoaromatic products from model diaromatics in the presence of limiting amounts of the cosubstrates NAD^+^ and GSH. The ability to recycle NAD^+^ and GSH allows the use of small quantities of these expensive cofactors and increases the future utility of a coupled enzyme system for processing lignin oligomers *in vitro*. Finally, we showed that this coupled enzyme system has activity with fractionated lignin oligomers. Below we summarize the new information gained from using this assay with widely used or new model β-ether linked substrates as well as lignin oligomers of different sizes.

### Insights gained from using the coupled assay with diaromatic compounds.

Using GGE as a substrate, we demonstrated that the GSH reductase AvGR is capable of recycling the cosubstrates NAD^+^ and GSH, enabling the β-etherase enzymes to completely cleave GGE in the presence of substoichiometric amounts of these cofactors (Fig. 2A to C). The A. vinosum DSM180 AvGR is well suited for this purpose, as most glutathione reductases described in the literature use NADPH instead of NADH as an electron donor (33). When AvGR was not present in an assay in which GGE concentrations were greater than those of NAD^+^ and GSH, there was incomplete hydrolysis of this diaromatic substrate, accumulation of β-etherase pathway intermediates, and depletion of NAD^+^, as expected if the reaction was cofactor limited.

We were also able to detect the release of tricin when GTE was used as a substrate in this assay, showing that β-etherase pathway enzymes are capable of β-ether bond cleavage in a substrate bearing a large flavonoid moiety. This further shows that the β-etherase pathway enzymes are not limited to substrates containing only G and S monoaromatic units. In prior research, we had demonstrated the ability of LigE and LigF to cleave G–G, G–S, S–G, and S–S dimer models (17, 21), so this result extends the knowledge of the diversity of substrates for these enzymes to the G–T dimers. Thus, although the β-etherase pathway enzymes are thought to be highly stereospecific, they are also capable of recognizing the many different configurations of β-ether-linked aromatics potentially present in lignin. With the results of these and previous findings combined (17, 21), we conclude that the minimal set of enzymes used in this study is sufficient to enable the β-etherase pathway *in vitro* to release G, S, and T units from compounds modeling β-ether units in lignin.

This coupled assay also allowed us to directly compare the abilities of LigG and NaGST_NU_ to function in the β-etherase pathway. We found that the presence of NaGST_NU_ and AvGR, along with LigD, LigN, LigE, and LigF, was sufficient to allow complete conversion of GGE to HPV and guaiacol (Fig. 2A to C). This is consistent with our prediction that NaGST_NU_ can accommodate both GS-HPV epimers in its active site (Kontur et al., submitted) and the ability of this enzyme to produce stoichiometric amounts of HPV from GGE when added to this coupled assay. In contrast, when LigG replaced NaGST_NU_ under otherwise identical assay conditions, there was incomplete hydrolysis of GGE to HPV and guaiacol, with significant accumulation of GGE-ketone and smaller amounts of GS-HPV (Fig. 2A, D, and E). Thus, although it has been suggested that LigG can hydrolyze both β-epimers of GS-HPV (24), this result, along with those published previously (21), supports the hypothesis that LigG has a strong preference for β(*R*)-GS-HPV. This direct comparison of substrate conversion to products in assays that differ only in the addition of LigG or NaGST_NU_ allows us to conclude that use of the latter enzyme has advantages owing to its greater ability to release HPV from both GS-HPV epimers under comparable conditions *in vitro*.

### Release of aromatic monomers from lignin oligomers *in vitro*.

The features of this coupled β-etherase assay allowed us to begin testing the ability to remove monomer aromatics from bona fide lignin. Lignin is a heterogeneous, high-molecular-weight polymer, with only limited solubility under the aqueous buffer conditions used for this assay. Consequently, to increase our chances of observing aromatic products under the conditions used for the coupled assay, we used several different lignin oligomers. We also fractionated these materials to test for release of aromatics from different-size lignin oligomers. This has provided several important new insights into the activity of β-etherase enzymes with lignin oligomers and identified opportunities for increasing our understanding of this pathway.

We tested the ability of this enzyme mixture to cleave lignin oligomers that were derived from HP lignin, an engineered poplar line comprised of as much as 97.5% S units (29). HPS was detected as a product when high-molecular-weight fractions of the HP lignin were used as the substrate. This provides direct proof that the enzyme mixture cleaves aromatic oligomers containing S units and that this set of β-etherase pathway enzymes is active with lignin oligomers. Given that the vast majority of the aromatic units in HP lignin are S units (29), we estimate that the oligomers used in the enzymatic assay had between 40 and 50 aromatic units (Table 1). With the concentration of lignin oligomer used in this assay (∼2.2 mg ml^−1^), complete substrate degradation would yield ∼8 mM HPS. The measured HPS concentration in this assay was 1.0 mM, resulting in a 12.5% yield of HPS from HP lignin. Thus, it appears that the mixture of enzymes used in this study, while sufficient for complete cleavage of model diaromatic compounds and of some β-ether links in HP lignin, is not capable of complete cleavage of all the β-ether linkages in the HP lignin oligomers. It is possible that a heretofore-undescribed protein is required to further process these lignin oligomers or that inhibition of enzyme activity was caused by the presence of some of the high-MW oligomers. Although our findings with the model dimers, and previous research, indicate that LigD and LigN are sufficient for complete oxidation of diaromatic compounds (Fig. 2) (19, 34), it is possible that the seemingly redundant dehydrogenases LigO and LigL have a higher affinity for higher-MW lignin oligomers. Similarly, LigP, a GSH-S-transferase with apparent redundant activity with LigE (20), may be of interest for the optimization of *in vitro* lignin depolymerization.

In the assays using HP lignin as a substrate, we did not detect syringaresinol as a product, even though this dimer is found in moderate abundance in this polymer (29). Existing models for the composition of HP lignin predict that syringaresinol is primarily internal to the polymer (29). Thus, it is possible that the failure to detect syringaresinol as a reaction product reflects the inability of the tested β-etherase enzymes to access and cleave β-ether bonds that are adjacent to a syringaresinol moiety or, perhaps, that the enzymes exhibited only limited exolytic activity, thus preventing the enzymes from ever reaching syringaresinol units in the polymer.

Having established that the coupled enzymatic assay exhibited β-etherase catalytic activity with high-MW fractions of the HP lignin oligomers, we tested a more complex lignin sample from corn stover as a substrate (MCS lignin). This lignin has been thoroughly characterized and shown to contain only tricin, which is covalently bonded to the lignin oligomers (9). Fractionation of this lignin was carried out and experiments with a wider array of lignin fractions were conducted to test for the release of the major aromatic monomers present in this material (G, S, and T units). Unfortunately, background absorbance was observed in the chromatograms, for all lignin fractions analyzed, at retention times that overlapped with tricin's. Although this background absorbance was present in all lignin fractions, fortunately, tricin was detectable as a defined absorbance peak with a retention time identical to that from the pure standard. The detection of HPV, HPS, and tricin from different MCS lignin fractions confirms the observations with the β-ether-linked models that the enzyme set used was active in the release of G, S, and T units from lignin. However, tricin was observed only with the lignin fraction having an average MW of 460 (Fig. 7). Using a crude assumption that the average aromatic unit in lignin has an MW of 210 and the known MW of tricin (330), this fraction represents mostly lignin dimers or a T unit with at most one or two other S or G units. Thus, the ability of the enzymes to cleave the β-ether linkage next to a flavonoid moiety appears to be restricted to lower-MW oligomers. In contrast, HPS and HPV were released from MCS lignin in assays using all of the fractions tested (Fig. 8), which we estimate to encompass a range of oligomers from dimers to 50-unit oligomers (Table 2). The highest measured concentration of HPS and HPV corresponded to the lignin fraction with an average MW of 1,390, or ∼7 aromatic units (Table 2). Using the same assumption of 210 as the average MW of an aromatic unit in lignin, and the mass of lignin used in the assay (2.2 mg ml^−1^), we estimate a yield of HPS plus HPV of ∼5%, which is lower than the estimated HPS yield from HP lignin. This lower release of substrates from MCS than HP lignin likely reflects the more heterogeneous and complex structure of the MCS lignin sample and potential inability of the β-etherase pathway enzymes to access and cleave all β-ether bonds in the polymer. The low yield of low-MW aromatics in the multistep enzymatic study (25) is in agreement with the low release of HPS and HPV observed in this study, supporting the hypothesis that the currently known β-etherase enzymes are not sufficient for complete breakdown of β-ether bonds in polymeric lignins.

Taken together, the findings presented here reveal new and exciting features of the β-etherase pathway enzymes. We identified tricin as a valuable flavonoid that can be enzymatically cleaved from β-ether-linked models and from low-MW lignin fractions. We also demonstrated β-etherase activity with intact lignin oligomers of various sizes, some of which might even be too large to be transported into cells. These findings therefore provide a demonstration that partial *in vitro* depolymerization of lignin is possible with β-etherase enzymes, an important step toward the development of biotechnological applications designed to derive high-value monomeric compounds from bona fide lignin polymers. Improving *in vitro* lignin depolymerization may depend on future discoveries of novel enzymes active on β-ether bonds, such as the newly characterized NaGST_NU_ (Kontur et al., submitted) used in this study, or the engineering of existing β-etherase enzymes to improve the range of β-ether-containing substrates they can utilize. Ultimately, the activity of the studied enzymes on oligomeric substrates provides an opportunity to develop and optimize conditions for aromatic release from lignin fractions derived from biomass deconstruction chemistries that are or will be used by industry.

## MATERIALS AND METHODS

### General.

GGE was purchased from TCI America (Portland, OR). Tricin, GTE, GTE-ketone, HPV, γ-hydroxypropiosyringone (HPS), and GGE-ketone were synthesized by previously described methods (7, 9, 35). All other chemicals were purchased from Sigma-Aldrich (St. Louis, MO). Methods to isolate and thoroughly characterize maize (*Zea mays*) corn stover (MCS) and high-syringyl transgenic hybrid poplar (HP) lignin samples, including gel permeation chromatography (GPC) and nuclear magnetic resonance (NMR) analyses and interpretation, were described previously (9, 29, 30). Manipulation of DNA and preparation of Escherichia coli transformant cultures were carried out according to previously described methods (36). All *lig* genes from Sphingobium sp. strain SYK-6, as well as the gene encoding AvGR from A. vinosum DSM180, were codon optimized for expression in E. coli and obtained from GeneArt (Life Technologies). NaGST_NU_ was amplified and cloned from N. aromaticivorans DSM12444 genomic DNA (Kontur et al., submitted).

### Plasmid and protein preparation.

Procedures for cloning, recombinant expression, and purification of Tev protease, LigE, LigF, LigG, and NaGST_NU_ are described elsewhere (21; Kontur et al., submitted). *ligD*, *ligN*, and the gene for AvGR, all codon optimized, were cloned into plasmid pVP302K (21) via the PCR overlap method (37–40). Expression and purification of LigD, LigN, NaGST_NU_, and AvGR followed procedures similar to those used previously (21). Briefly, E. coli strain B834 cultures, transformed with expression plasmids, were grown aerobically overnight in 1 liter of autoinduction ZYM-5052 medium (41) supplemented with 100 μg ml^−1^ of kanamycin. Cells were pelleted and extracts prepared via compression and sonication. Histidine-tagged proteins were purified from cell lysates via nickel-nitrilotriacetic acid (Ni-NTA) affinity chromatography with Qiagen Ni-NTA resin. His-tagged Tev protease was used to liberate N-terminal His tags and a second round of Ni-NTA affinity chromatography was used to remove the tag and Tev protease before separation by size exclusion chromatography. Protein preparations were concentrated and frozen with liquid N_2_.

### Enzyme assays.

*In vitro* enzyme assays with LigD, LigN, LigE, LigF, NaGST_NU_ (or LigG), and AvGR (or a subset of those enzymes) were conducted in assay buffer (25 mM Tris, 2.0% dimethyl sulfoxide [DMSO] [pH 8.0]). The concentration of each enzyme was 50 μg ml^−1^ in all assays. When GGE (6 mM) was the substrate, the initial cosubstrate concentrations were 2 mM NAD^+^ and 4 mM GSH. When GTE (1 mM) was the substrate, the initial cosubstrate concentrations were 5 mM NAD^+^ and 5 mM GSH. When an isolated lignin sample was used as the substrate (2.2 mg ml^−1^), the initial cosubstrate concentrations were 2 mM NAD^+^ and 4 mM GSH. Enzyme assays (volume, 1 ml or larger as needed) were carried out (in duplicate) in 2-ml vials that received the following in sequential order: (i) 20 μl of substrate (GGE, GTE, or lignin) dissolved in DMSO (concentrated 50 times above the intended assay concentration), (ii) 880 μl of 25.6 mM Tris (pH 11.5) (where the acidic effect of GSH drops the pH to 8.0 after addition of 5 mM GSH), (iii) 50 μl of a stock solution in 25 mM Tris containing NAD^+^ and GSH (each is concentrated 20 times above the intended assay concentration), and (iv) 50 μl of a 20-times-concentrated mixture of the desired enzymes. At the desired time points, 150-μl samples were removed from an assay and enzymatic activity was abolished by pipetting into 5 μl of 5 M phosphoric acid. GGE, guaiacol, HPV, and HPS concentrations were quantified for each time point (see below) using a linear regression of known standards for each compound.

### GPC. (i) Preparative GPC.

GPC of lignin samples was carried out using a Beckman 125NM solvent delivery module equipped with a Beckman 168 UV detector (λ = 280 nm) and a 30-ml Bio-Rad Bio Bead S-X3 column (a neutral, porous styrene-divinylbenzene copolymer). Dimethylformamide (DMF) was used as the mobile phase at a flow rate of 1.0 ml min^−1^. Between 20 and 50 mg of lignin was dissolved in a minimal amount of DMF and injected into the mobile phase, and 1-ml fractions were collected until UV absorption decreased to baseline levels. Fractions were then subjected to analytical GPC to estimate their average molecular weight (MW). The DMF was evaporated *in vacuo* in order to recover material used for enzyme assays.

### (ii) Analytical GPC.

Analytical GPC of lignin samples was carried out with a Shimadzu Prominence ultrafast liquid chromatography system (LC-20AD pumps, SIL-20AC HT autosampler, CTO-20A column oven, and CBM-20A controller) and using two TSKgel Alpha-2500 (300 by 7.8 mm; Tosoh Bioscience) columns at 40°C. Samples (10-μl injection volume) containing approximately 1 mg ml^−1^ of isolated or GPC-fractionated lignin were injected into a mobile phase (100 μM LiBr in DMF) at a flow rate of 0.3 ml min^−1^ with a run length of 90 min. An SPD-M20A photodiode array detector (λ = 200 nm) was used for the determination of elution times, which were subsequently converted to MWs using regression analysis of ReadyCal kit polystyrene standards.

### C_18_ column chromatography.

C_18_ column chromatographic separations were carried out using a Beckman 125NM solvent delivery module equipped with a Beckman 168 UV detector. Samples of 150 μl were collected from enzyme assays and 20-μl aliquots were injected into either a 4- by 120-mm Restek Ultra aqueous C_18_-reversed stationary-phase column or a 4.6- by 250-mm Phenomenex Luna 5u C_18_(2)-reversed stationary-phase column with a 1.0-ml min^−1^ mobile phase composed of a mixture of an aqueous buffer (5 mM formic acid in 95% H_2_O–5% acetonitrile) and methanol. Samples from enzyme assays using GTE as the substrate were analyzed on the Phenomenex column to improve separation of GTE and tricin. All other C_18_ column chromatographic separations were carried out using the Restek column. For the Restek column, the methanol fraction of the buffer (with aqueous buffer as the remainder) was adjusted as follows: 0 to 6 min, 30% methanol; 6 to 15 min, gradient from 30 to 80% methanol; 15 to 27 min, 80% methanol; 27 to 28 min, gradient from 80 to 30% methanol; and 28 to 33 min, 30% methanol. For the Phenomenex column, the gradient system was as follows: 0 to 6 min, 10% methanol; 6 to 50 min, gradient from 10 to 90% methanol; 50 to 63 min, 90% methanol; 63 to 64 min, gradient from 90 to 10% methanol; and 64 to 70 min, 10% methanol.

## Supplementary Material

Supplemental material
