# Supplementary material for: In Vitro Enzymatic Depolymerization of Lignin with Release of Syringyl, Guaiacyl, and Tricin Units
Source: Appl Environ Microbiol. 2018 Jan 17;84(3):e02076-17. doi: 10.1128/AEM.02076-17 (PMC5772236; doi:10.1128/AEM.02076-17)
Supplement: Supplemental material [file supp_84_3_e02076-17__index.html]

Supplemental material 

# *In Vitro* Enzymatic Depolymerization of Lignin with Release of Syringyl, Guaiacyl, and Tricin Units

## Supplemental material

- Supplemental file 1 -

  Summary of *in vitro* reactions performed in this study (Table S1).

  PDF, 178K
