# Supplementary material for: In Vitro Enzymatic Depolymerization of Lignin with Release of Syringyl, Guaiacyl, and Tricin Units
Source: Appl Environ Microbiol. 2018 Jan 17;84(3):e02076-17. doi: 10.1128/AEM.02076-17 (PMC5772236; doi:10.1128/AEM.02076-17)
Supplement: Supplemental material [file AEM.02076-17_zam003188296s1.pdf]

Supplementary Information for:

In vitro enzymatic depolymerization of lignin with release of syringyl, guaiacyl, and tricin units

Daniel L. Gall,[1] Wayne S. Kontur,[1] Wu Lan,[1,2] Hoon Kim,[1,2] Yanding Li,[1,2] John Ralph,[1,2] Timothy J. Donohue,[1,3] and Daniel R. Noguera[1,4]

[1]Great Lakes Bioenergy Research Center, Wisconsin Energy Institute, University of Wisconsin, Madison WI 53726

[2]Department of Biochemistry, University of Wisconsin, Madison, WI 53706

[3]Department of Bacteriology, University of Wisconsin, Madison, WI 53706

[4]Department of Civil & Environmental Engineering, University of Wisconsin, Madison, WI 53706

Corresponding author: Noguera, Daniel R. (dnoguera@wisc.edu)

Table S1. Summary of *in vitro* reactions performed in this study

<table>
<tr><th>Reaction</th><th>Enzymes</th><th>Initial Substrate Concentrations</th><th>Final Substrate Concentrations</th><th>Products</th></tr>
<tr><td>1.
(Fig. 2B)</td><td>LigD, LigN, LigE, LigF, NaGST$_{NU}$</td><td rowspan="4"><i>Erythro</i>-GGE, 6.0 mM
NAD$^+$, 2 mM
GSH, 4 mM</td><td><i>Erythro</i>-GGE, 3.8 mM
NAD$^+$, 0 mM[a]</td><td>HPV, 2.0 mM
Guaiacol, 2.0 mM
Threo-GGE, 0.1 mM</td></tr>
<tr><td>2.
(Fig. 2C, Fig. 3)</td><td>LigD, LigN, LigE, LigF, NaGST$_{NU}$, AvGR</td><td><i>Erythro</i>-GGE, 0 mM
NAD$^+$, 2 mM[a]</td><td>HPV, 6.1 mM
Guaiacol, 5.9 mM</td></tr>
<tr><td>3.
(Fig. 2D)</td><td>LigD, LigN, LigE, LigF, LigG</td><td>Erythro-GGE, 3.9 mM
NAD$^+$, 0 mM[a]</td><td>HPV, 2.0 mM
Guaiacol, 2.0 mM
Threo-GGE, 0.1 mM</td></tr>
<tr><td>4.
(Fig. 2E)</td><td>LigD, LigN, LigE, LigF, LigG, AvGR</td><td>Erythro-GGE, 0.02 mM
NAD$^+$, 2 mM[a]</td><td>HPV, 5.3 mM
Guaiacol, 5.2 mM
Threo-GGE, 0.02 mM
GS-HPV[b]
GGE-ketone, 0.7 mM</td></tr>
<tr><td>5.
(Fig. 4B)</td><td>LigD, LigN, LigE, LigF, NaGST$_{NU}$</td><td rowspan="3"><i>Erythro</i>-GTE, 0.86 mM
Threo-GTE, 0.14 mM
NAD$^+$, 5 mM
GSH, 5 mM</td><td><i>Erythro</i>-GTE, 0 mM
Threo-GTE, 0 mM
NAD$^+$, 5 mM[a]</td><td>HPV, 1.1 mM
tricin[b]</td></tr>
<tr><td>6.
(Fig. 4C)</td><td>LigD, LigN, LigE, LigF</td><td>Erythro-GTE, 0 mM
Threo-GTE, 0 mM
NAD$^+$, 4 mM[a]</td><td>tricin[b]
GS-HPV[b]</td></tr>
<tr><td>7.
(Fig. 4D)</td><td>LigD, LigN</td><td>Erythro-GTE, 0 mM
Threo-GTE, 0 mM
NAD$^+$, 4 mM[a]</td><td>GTE-ketone[b]</td></tr>
<tr><td>8.
(Fig. 6B)</td><td>LigD, LigN, LigE, LigF, LigG, AvGR</td><td>HP lignin, 2.2 mg/mL
NAD$^+$, 2 mM
GSH, 4 mM</td><td>HP lignin[c]
NAD$^+$, 2 mM[a]</td><td>HPS, 1.0 mM
unknown</td></tr>
<tr><td>9.
(Fig. 8B)</td><td rowspan="5">LigD, LigN, LigE, LigF, NaGST$_{NU}$, AvGR</td><td>MCS lignin fraction 1 (MW 10,710), 2.2 mg/mL
NAD$^+$, 2 mM
GSH, 4 mM</td><td>MCS lignin[c]
NAD$^+$, 2 mM[a]</td><td>HPV, 0.02 mM
HPS, 0.01 mM
unknowns</td></tr>
<tr><td>10.
(Fig. 8C)</td><td>MCS lignin fraction 5 (MW 5,370), 2.2 mg/mL
NAD$^+$, 2 mM
GSH, 4 mM</td><td>MCS lignin[c]
NAD$^+$, 2 mM[a]</td><td>HPV, 0.04 mM
HPS, 0.02 mM
unknowns</td></tr>
<tr><td>11.
(Fig. 8D)</td><td>MCS lignin fraction 8 (MW 1,390), 2.2 mg/mL
NAD$^+$, 2 mM
GSH, 4 mM</td><td>MCS lignin[c]
NAD$^+$, 2 mM[a]</td><td>HPV, 0.4 mM
HPS, 0.1 mM
unknowns</td></tr>
<tr><td>12.
(Fig. 8E)</td><td>MCS lignin fraction 14 (MW 660), 2.2 mg/mL
NAD$^+$, 2 mM
GSH, 4 mM</td><td>MCS lignin[c]
NAD$^+$, 2 mM[a]</td><td>HPV, 0.3 mM
HPS, 0.1 mM
unknowns</td></tr>
<tr><td>13.
(Fig. 8F)</td><td>MCS lignin fraction 17 (MW 460), 2.2 mg/mL
NAD$^+$, 2 mM
GSH, 4 mM</td><td>MCS lignin[c]
NAD$^+$, 2 mM[a]</td><td>HPV, 0.3 mM
HPS, 0 mM
unknowns</td></tr>
</table>

[a] Reported NAD$^+$ concentrations are approximations based on peak area and initial concentrations.

[b] GS-HPV, tricin, and GTE-ketone concentrations could not be quantified due to lack of sufficient standards.

[c] HP and MCS lignin, at the end of the enzymatic reaction, could not be quantified due to the small quantities used in the assays.
